# Circulating Oxidized Low-Density Lipoproteins and Antibodies against Oxidized Low-Density Lipoproteins as Potential Biomarkers of Colorectal Cancer

**DOI:** 10.1155/2015/146819

**Published:** 2015-03-31

**Authors:** Dorota Diakowska, Krzysztof Grabowski, Mirosław Nienartowicz, Paweł Zarębski, Kamila Fudalej, Krystyna Markocka-Mączka

**Affiliations:** Department of Gastrointestinal and General Surgery, Wroclaw University of Medicine, Sklodowskiej-Curie 66, 50-367 Wroclaw, Poland

## Abstract

*Introduction*. The aim of the study was evaluation of the diagnostic utility of serum oxidized low-density lipoproteins (oxLDL), antibodies against oxLDLs (o-LAB), and CEA as risk markers of colorectal cancer (CRC). *Material and Methods*. The serum levels of study factors were measured in 73 patients with CRC and in 35 healthy controls who were gender- and BMI-matched to the study group. Concentrations of oxLDL, o-LAB, and CEA were detected in ELISA tests. Serum lipids, lipoproteins, and glucose levels were also coestimated. *Results*. Age and o-LAB were significant factors of CRC presence, but results of logistic regression analysis showed that both were weak predictors of CRC risk. Concentration of o-LAB was significantly higher in colon cancer than in rectal cancer, especially when the cancer was located in the right section of colon. Serum CEA levels were significantly elevated in the advanced stage of disease, primary tumor progression, angiolymphatic invasion, and presence of distant metastasis. *Conclusions*. Obtained results have demonstrated that oxLDL and o-LAB were not satisfactory risk markers of CRC. Although significant relation between o-LAB level and CRC is observed, it may be rather the result of individual differences in the host immune responses against cancer.

## 1. Introduction

Colorectal cancer (CRC) ranks among the most frequent malignancies in Poland. It is the second most common cancer among men (after lung cancer) and the third among women (after lung and breast cancers). CRC is the second leading cause of cancer-related death for man and the third for woman in Poland [1]. Potential prognostic and predictive biomarkers of CRC are still investigated. Identification of these factors, especially in patient's blood, may play an important role in prevention, screening, and treatment improvement of CRC [2].

It has been demonstrated that carcinoembryonic antigen (CEA) is an important biomarker of CRC, which may be used in clinical practice [3]. However, earlier studies demonstrated that serum CEA proves limitation of sensitivity for early diagnosis of CRC. Therefore CEA is not recommended as a screening factor for CRC, but it may be a marker for monitoring of surgical treatment efficiency and systemic therapy [2, 4, 5].

It has been already well documented that oxidative stress is a promutagenic and procarcinogenic process [6, 7]. Products of lipids oxidation formed from blood lipids and lipoproteins, which determine the intensity of oxidative stress, are studied as potential markers of CRC development [7–13].

Findings from many studies, which have evaluated the role of total cholesterol (TC) and triglycerides (TG) as risk factors in CRC development, have been inconsistent [7, 8]. Some studies have indicated a positive association between TC, TG, and CRC [8–10], but other researches have observed insignificant relations between these molecules and CRC risk [11, 12]. Additionally, the positive relation between blood glucose and CRC was observed [12]. Data concerning serum high density lipoprotein cholesterol (HDL-C) and low-density lipoprotein cholesterol (LDL-C) in the development of this type of cancer remain unexplained [7–11]. HDL-C molecules display antioxidative activities, stimulate production of anti-inflammatory cytokines, and act as anticarcinogens [8, 11]. Unlike HDL-Cs, LDL-Cs are susceptible to oxidation and generation of oxidized low-density lipoprotein (oxLDL) products [6, 7, 13]. It was observed in vascular cells that LDL oxidation stimulates overproduction of reactive oxygen species, proinflammatory proteins, and biologically active molecules with promutagenic activity [7].

oxLDLs are taken up by macrophages and antibodies against oxLDLs, named o-LAB. The o-LAB plays a positive role in reduction of oxLDL concentration in serum [13]. Our previous studies [14] hypothesize that lower levels of serum o-LAB may be related to esophageal cancer progression.

The role of circulating oxLDL and o-LAB as possible prognostic markers in CRC remains unclear. Therefore, the aim of this study was to assess the concentrations of oxLDL and o-LAB in serum of patients with colorectal cancer. The relationships between levels of these factors and clinic-pathological parameters in CRC were analyzed. Serum concentration of CEA and serum levels of glucose, total cholesterol, triglycerides, and lipoproteins fractions were also coestimated. Possible correlations between study factors were investigated.

## 2. Material and Methods

### 2.1. Patients Characteristic

A group of 73 patients with CRC, who were admitted between 2008 and 2013 to the Department of Gastrointestinal and General Surgery of Wroclaw University of Medicine for curative resection of histopathologically confirmed adenocarcinoma of the colon or rectum, has been selected for the study. Patients with prior radio- or chemotherapy were excluded from the study. Also patients with any systemic illness were not rated among the examined group. Study group consists of 44 male and 29 female, median age 70 years (interquartile range: 58–76). The majority of primary tumors were located in the colon (*n* = 52, 71.2%); tumor in rectum was placed in 21 patients (28.8%). Open colectomies (right hemicolectomy *n* = 23, transverse colectomy *n* = 9, left hemicolectomy *n* = 3, and sigmoidectomy *n* = 17) were performed. Resected tumors were staged pathologically, according to the UICC TNM staging system [15]. There were 7 cases with stage I, 35 with stage II, 20 with stage III, and 11 with stage IV. Histological differentiation was well in 8 patients, moderate in 62 patients, and poor in 3 patients.

The control group included 35 healthy blood donors (23 male, 12 female) with median age 57 years (interquartile range: 55–62). Control group was gender- and BMI-matched to study group, but age difference between study and control group was statistically significant, *P* < 0.001 (Table 1). Age parameter was a confounding factor in this study, which had to be taken into account in the statistical analyses of data.

### 2.2. Ethical Considerations

The study was planned according to the ethical standards detailed in the Declaration of Helsinki, as revised in 1983. The study protocol was approved by the Medical Ethics Committee, University of Medicine, Wroclaw, Poland. Informed consent has been obtained from all subjects.

### 2.3. Analytical Methods

Peripheral blood samples were obtained from controls and CRC patients (before surgery) into sterile vacuum tubes. Blood was clotted (30 min., RT) and centrifuged (1500 ×g, 10 min., RT). Each serum sample was divided into three Eppendorf tubes (300–600 *μ*L/tube) which were afterwards stored at −45°C until the appropriate analysis.

Levels of oxLDL were assayed by solid phase two-site ELISA test (Mercodia, Uppsala, Sweden), in which two monoclonal antibodies are directed against separate antigenic determinants on the oxidized apolipoprotein B-100 (apoB-100). During the first incubation oxLDLs in the sample react with capture murine monoclonal antibodies mAb-4E6, which coat the wells of microtiter plate. After wash, when nonreactive components are removed, peroxidase conjugated anti-human apoB-100 antibodies recognize the oxLDLs, which are bound to the solid phase. Measurements of oxLDL levels in serum samples were performed every six months, as recommended in manufacture's instruction of the test. Each serum sample has been thawed only once and has been tested in duplicate. Obtained results of few tests, which were performed between 2008 and 2013, were kept in database.

Serum o-LAB was determined by ELISA kit (Biomedica, Vienna, Austria). Molecules of o-LAB in the sample react with oxidised LDLs, which coat microtiter strips as antigens. Normal range of test was 119–324 U/L.

CEA concentration was determined by CanAg CEA EIA kit (Fujirebio Diagnostics AB, Goteborg, Sweden). Measurement range of test was 0.25–75 *μ*g/L.

Concentrations of TC, HDL-C (direct test), LDL-C (direct test), TG, and Glu were measured spectrophotometrically with using the enzymatic methods. All used kits were produced by BioMaxima company (BioMaxima, Lublin, Poland). All samples were tested in duplicate in the above mentioned kits.

Data concerning patient's body mass index (BMI), weight (kg)/height^2^ (m), were collected from case histories. BMI of healthy blood donors was calculated from short interview concerning their weight and height.

### 2.4. Statistical Analysis

Distribution of data was analyzed by the Shapiro-Wilk normality test and equality of variances by Levene's test. Concentrations of biochemical parameters were presented as medians and interquartile ranges Q25–Q75. In dependence on data distribution and homogeneity of variances, differences between groups were analyzed by Student's *t*-test, one-way ANOVA and post hoc Tukey test or Mann-Whitney test, Kruskal-Wallis test, and post hoc Dunn's test. Frequency analysis was conducted by chi-square test. Single correlations were performed by Pearson test or Spearman's rank correlation test.

Analysis of covariance (ANCOVA) was used for evaluation of influence of examined variables on o-LAB level. In this analysis, o-LAB level was a dependent variable, and age parameter was a confounding factor. Stepwise method of multiple regression analysis (with *P* < 0.05 as an entrance criterion and *P* > 0.1 as a removal criterion) was conducted in order to confirm ANCOVA results.

Multiple logistic regression was used for determination of independent predictors of CRC presence (criteria were the same as for multiple regression). ROC analysis was used to determine the cut-off points for predictors of CRC presence. Cut-off points for studied factors allowed the differentiation of two groups (cancer/control) with the highest sensitivity and specificity.

All values of *P* < 0.05 were considered as statistically significant. The statistical analyses were performed using STATISTICA 10.0 software (StatSoft, Inc., Tulsa, OK, USA).

## 3. Results

### 3.1. Demographic and Biochemical Characteristics of CRC Patients in Comparison to Healthy Controls. The Role of Age and o-LAB Factors as Predictors of CRC Presence

As showed in Table 1, demographic and biochemical characteristics of CRC patients and the controls demonstrated no differences except for o-LAB level and age parameter (both significantly higher in CRC patients). To evaluate the influence of age on o-LAB level we applied ANCOVA analysis, with cancer (0/1) as an independent factor and with age as a confounding factor (Table 2). Levels of o-LAB were significantly influenced by cancer presence (*P* < 0.0001). Age increase had an insignificant influence on o-LAB levels (*P* = 0.102). Also multiple regression analysis confirmed lack of association between age parameter and o-LAB concentration (Table 2). It implies that age does not affect o-LAB levels in CRC presence.

Age and o-LAB parameters, as independent, associated with the presence of CRC variables, were identified in logistic regression analysis (Table 3). Advanced age and high o-LAB levels turned out to be predictors of cancer presence in 73.7% of cases (overall model fit: chi^2^ = 47.4, *P* < 0.0001). On the other hand odds ratio values calculated for o-LAB (OR = 1.0) and age (OR = 1.2) showed that these factors were weak predictors of CRC. ROC analysis demonstrated that cut-off point for o-LAB level was 842 U/L. Remaining parameters of ROC analysis were AUC = 0.698; 95% CI: 0.595–0.801; *P* < 0.028; sensitivity: 30.1%; specificity: 96.2%. It was also calculated that age above 63 years was related to development of CRC: AUC = 0.870; 95% CI: 0.800–0.940; *P* < 0.001; sensitivity: 78%; specificity: 96%.

### 3.2. oxLDL and o-LAB Association with Location and Tumor Grade of CRC

Statistically significant relationship between o-LAB level and location of primary tumor in CRC was found. The levels of o-LAB were significantly higher in subgroup of patients with colon cancer than in patients with rectal cancer (Table 1). We demonstrated that serum levels of TC and HDL-C were also significantly higher in colon cancer in comparison to rectal cancer. ANCOVA analysis showed that localization of primary tumor in colon or rectum (*P* = 0.003) but not TC (*P* = 0.094) and HDL-C (*P* = 0.355) was significantly associated with serum o-LAB. Multiple regression analysis confirmed that only o-LAB was related to location of CRC in colon or rectum (overall: *R* ^2^ = 0.25, *P* < 0.001; *β* coefficient for o-LAB = 0.32, *P* = 0.003). Data in Table 4 showed that the highest concentrations of o-LAB were in cases of tumor location in the right colon and the lowest levels of o-LAB were in location of the tumor in the rectal segment (*P* = 0.023).

As demonstrated in Table 4, the concentrations of serum o-LAB were significantly higher in CRC with T1+2 in comparison with T4 primary tumor (*P* < 0.001). Also levels of serum oxLDL were significantly higher in CRC patients with T1+2 primary tumor than in patients with T3 or T4 tumor stage (*P* = 0.044 and *P* = 0.021, resp.). However size of subgroup of T1+2 patients was too low (*n* = 11) and these results should be tested on larger group of CRC patients.

### 3.3. CEA Marker in CRC Patients

It was interesting that levels of CEA marker in the controls, total CRC group, and subgroups of CRC patients were not significantly different (Table 1). Analysis of the serum CEA concentrations in relation to clinical and pathological parameters of cancer patients (data in Table 4) revealed no significant differences in age, gender, histological differentiation, and tumor location. However, high serum CEA levels were significantly associated with stage of disease (*P* = 0.004), primary tumor progression (*P* = 0.002), angiolymphatic invasion (*P* = 0.005), and distant metastasis (*P* = 0.032).

### 3.4. Correlations between Study Parameters in CRC Patients

We observed the strongest positive correlations between following factors: TC and LDL-C (rho = 0.727, *P* < 0.001), BMI and TC (rho = 0.669, *P* < 0.001), BMI and LDL-C (rho = 0.554, *P* < 0.001), LDL-C and TG (rho = 0.485, *P* < 0.001), and oxLDL and Glu (rho = 0.388, *P* = 0.009).

Significant positive correlation between oxLDL and o-LAB levels (rho = 0.384, *P* < 0.006) and significant negative association between CEA and o-LAB concentrations (rho = −0.252, *P* = 0.031) were demonstrated in correlation analysis. Although probability values for both analyses were significant, statistical power of correlation coefficients was low. The negative relationship between CEA and o-LAB levels was also confirmed by segregation of CRC patients according to CEA concentration into two groups: with CEA < 5.0 *μ*g/L and with CEA ≥ 5.0 *μ*g/L. Increase of CEA level above 5.0 *μ*g/L was significantly related to reduction of o-LAB concentration in CRC patients (*P* = 0.008).

## 4. Discussion

Prospective studies suggest the mechanistic overlap in the pathobiology of atherogenesis and carcinogenesis [6]. Chronic inflammation and oxidative stress induce angiogenesis in both, atherogenesis and carcinogenesis, although these processes are more intensive in cancer progression [6, 7, 16–18]. Oxidatively modified low-density lipoproteins have been shown as ones of the primary products of oxidation reactions. Oxidized LDLs stimulate synthesis of reactive oxygen species and induce production of proinflammatory cytokines, such as proangiogenic VEGF and growth factors, which contributed to new blood vessels formation [6, 19–22]. Experimental study of Zettler et al. [23] has showed that oxLDL may be an independent mitogenic factor, which induces cellular cycle of proteins. High expression of cell cycle-activating proteins may stimulate malignant cell growth [24].

Process of oxidative modification of LDLs takes place in the endothelium of the arterial wall and a certain amount of oxLDLs is released into the blood [7, 21]. A positive relationship between increased serum oxLDL concentration and risk of breast, pancreas, colon, or esophageal cancer has been demonstrated [7, 13, 14, 25].

In our study we did not find significant differences in serum oxLDL levels between total group of CRC patients and healthy control. However, we found a significantly higher level of oxLDL in patients with early stage of primary tumor (T1+2) in comparison with patients with advanced stage of primary tumor progression (T3 and T4).

Our results are in accordance with Jiang et al.'s theory [6]. They hypothesized that oxLDL at high concentration induces the release of cytotoxic reactive oxygen species, which cause injury of host cells. Then low concentration of oxLDL mediates cycle of reactions, which induces VEGF expression and angiogenesis process. In our study, high concentration of oxLDL in early tumor development and low oxLDL concentration in advanced tumor (similar to level in control group) confirmed this theory. However, due to the small strength of subgroup of cancer patients with T1+2 primary tumor progression, further researches are needed for better understanding of the role of oxLDL in CRC.

Autoantibodies o-LAB are produced against epitopes of oxidized LDLs, which was firstly showed in atherosclerotic lesions in humans [26]. Previous studies have showed divergent results concerning serum o-LAB concentrations in cancer patients. One of them has demonstrated that o-LAB was negative in association with development of esophageal squamous cell carcinoma [14, 21] and other ones observed positive relation with presence of breast cancer [7]. As shown in study by Suzuki et al. [13] the levels of o-LAB were not significantly associated with risk of colorectal cancer.

Our results are in accordance with Suzuki et al.'s studies. We found a significant increase of o-LAB level in patients with CRC in comparison with control. However results obtained in the logistic regression analysis for o-LAB were not entirely satisfactory. We suggest that o-LAB does not have a predisposition to be an independent marker of CRC presence. We considered, as Suzuki et al. [13], that individual differences in immune responses may attenuate the relation between o-LAB and CRC presence. We also observed that advanced age was slightly better related to CRC development. This finding confirmed earlier observations concerning an important role of age increase as a risk factor for this type of cancer [27–29].

We showed the highest level of o-LAB, as in case of oxLDL concentration, in patients with early T1+2 tumor growth. It suggests that these factors may be associated with early neoplastic transformation in CRC. Yet our observations were performed on too small group of patients and they warrant further studies. We also observed significantly higher concentration of o-LAB in colon cancer than in rectal cancer, especially in the localization of cancer on the right section of colon. If our results are confirmed by studies on larger group of patients, they might suggest that o-LAB level is probably determinable by tumor location in CRC.

Significant correlation between serum oxLDL and o-LAB in CRC was observed, but this association had a weak power. The physiological role of o-LABs is to remove oxLDLs from blood by creating soluble oxLDL-o-LAB complexes. The level of o-LAB in blood depends not only on synthesis and release of antibodies into circulation, but also on many biological factors, which affect the titer of autoantibodies, and on genetic control of o-LAB production [7]. For that reason o-LAB levels might reflect individual differences in immune response rather than in generation of o-LAB against oxLDL only [13].

We did not observe any significant differences in levels of TC, TG, HDL-C, LDL-C, and glucose between CRC patients and the controls. Our study was so designed that the controls were BMI-matched to CRC group. Obtained results showed that concentrations of serum oxLDL and o-LAB were independent values of serum lipoproteins, lipids, and glucose.

In the present study we observed insignificant differences in serum CEA level between total CRC patients and healthy control and between early (TNM stage I + II) CRC and control. Our study confirmed that CEA should not be used as a screening factor for colorectal cancer. This observation is in accordance with ASCO and EGTM recommendations [2, 5]. However, we showed statistically higher concentrations of CEA in CRC patients with advanced stages of disease (III and IV). It may suggest the utility of CEA as an independent prognostic factor for surveillance after operation and after chemotherapy.

Negative correlation with low power was observed between CEA and o-LAB levels. This result confirms disturbances in mechanisms of expression and regulation of these two factors in cancer development.

In conclusion, obtained results have demonstrated that serum oxLDL and o-LAB were not satisfactory risk markers of CRC. Although significant relation between o-LAB level and CRC is observed, it may be rather a result of individual differences in host immune responses against cancer.

## Figures and Tables

**Table 1:** Demographic, clinical, and biochemical parameters in healthy control and total CRC patients and in colon and rectum subgroups. Data are presented as median (interquartile range Q25–Q75).

	(A) Control (*n* = 35)	(B) Total CRC (*n* = 73)	(C) Colon (*n* = 52)	(D) Rectum (*n* = 21)	*P* value 1 (A versus B)	*P* value 2 (A versus C versus D)
Gender (M/F)	23/12	44/29	28/24	16/5	0.130	0.097
Age (years)	57 (55–62)^a^	70 (58–76)	70 (63–76)^a^	69 (67–76)^a^	<0.001^*^	<0.001^*^
BMI (kg/m^2^)	25.4 (23.7–26.9)	24.5 (23.1–26.1)	24.7 (23.6–26.2)	23.6 (21.9–25.8)	0.194	0.072
CEA (*µ*g/L)	2.2 (0.4–3.2)	1.6 (0.7–4.2)	1.6 (0.7–4.8)	1.7 (0.7–3.8)	0.214	0.490
oxLDL (U/L)	62.0 (48.0–67.3)	60.1 (48.4–84.1)	64.4 (51.2–88.1)	49.3 (46.6–69.1)	0.287	0.172
o-LAB (U/L)	282.0 (219.6–409.2)^b^	488.0 (286.0–1046.0)	578.6 (285.6–1131.1)^b,c^	352.8 (285.6–450.8)^c^	0.003^*^	0.002^*^
TC (mg/dL)	164.5 (138.3–185.0)	175.0 (148.0–194.0)	183.5 (156.0–196.5)^d^	148.0 (125.0–176.0)^d^	0.519	0.003^*^
HDL-C (mg/dL)	60.2 (55.7–65.0)^e^	56.0 (48.0–65.0)	58.5 (51.5–67.0)^f^	51.0 (40.0–59.0)^e,f^	0.316	0.004^*^
LDL-C (mg/dL)	103.0 (86.0–127.0)	119.0 (93.0–128.0)	117.0 (93.0–128.0)	120.0 (92.0–129.0)	0.206	0.450
TG (mg/dL)	154.0 (134.0–159.0)	134.0 (117.0–152.0)	137.0 (122.0–152.5)	123.0 (106.0–152.0)	0.151	0.331
Glu (mg/dL)	97.0 (95.0–101.0)	96.5 (86.0–110.0)	96.0 (86.0–108.0)	97.0 (94.0–120.0)	0.364	0.661

M/F: male/female; ^*^statistically significant; ^a^ *P* < 0.001 for A versus C and A versus D; ^b^ *P* = 0.0001 for A versus C; ^c^ *P* = 0.005 for C versus D; ^d^ *P* = 0.002 for C versus D; ^e^ *P* = 0.014 for A versus D; ^f^ *P* = 0.004 for C versus. D.

**Table 2:** ANCOVA and multiple regression analysis for o-LAB as a dependent variable (age parameter as a continuous covariate).

ANCOVA	*F*-test	*P* value	
Group (CRC versus control)	16.1	0.0001^∗^	
Age (years)	2.7	0.102	

Multiple regression analysis *R* ^2^ = 0.38, *P* < 0.001	*β*-coefficient	*t*-test	*P* value

Group (CRC versus control)	0.44	4.0	0.0001^∗^
Age (years)	0.18	1.7	0.102

^∗^Statistically significant.

**Table 3:** Logistic regression analysis (stepwise method) of factors related to CRC presence. Serum o-LAB and age as independent variables; *b*: regression coefficient; SE_*b*_: standard error of *b*; OR: odds ratio; 95% CI: 95% confidence interval.

Variables	*b*	SE_*b*_	OR	95% CI	*P* value
Age (years)	0.183	0.043	1.201	1.104–1.306	<0.0001^∗^
o-LAB (U/L)	0.005	0.001	1.005	1.002–1.008	0.0003^∗^

^∗^Statistically significant.

**Table 4:** Relationships between serum levels of oxLDL and o-LAB and clinicopathological characteristics of patients with CRC (*n* = 73). Data are presented as median (interquartile range Q25–Q75).

Parameter	oxLDL (U/L)	*P* value	o-LAB (U/L)	*P* value	CEA (*µ*g/L)	*P* value
Age (years):		0.226		0.748		0.051
<70 (*n* = 34)	63.7 (53.2–88.1)		474.6 (260.8–1013.9)		1.2 (0.1–2.5)	
≥70 (*n* = 39)	57.2 (47.6–79.2)		488 (300–1123.9)		2.7 (0.8–5.2)	
Gender:		0.779		0.672		0.880
Male (*n* = 44)	64.4 (47.6–84.1)		493.8 (292.8–1030)		2.1 (0.7–3.7)	
Female (*n* = 29)	54.5 (49.6–78.1)		450.8 (212.6–1054)		1.4 (0.8–5.2)	
Histological grade:		0.984		0.617		0.083
G1 (*n* = 8)	68.7 (43.3–97.9)		199.4 (146.8–1223)		1.0 (0.1–2.5)	
G2 (*n* = 62)	54.8 (48.4–82.6)		491.2 (310.4–1046)		1.7 (0.7–4.0)	
G3 (*n* = 3)	66.1 (63.7–68.5)		602.6 (359.2–604.4)		5.2 (2.6–6.8)	
Primary tumor:		0.247		0.027^*^		0.788
Right colon (*n* = 32)	68.5 (53.7–88.1)		774 (497–1148)^1^		1.5 (0.3–4.8)	
Left colon (*n* = 20)	59.5 (44.0–84.1)		448 (204–1131)		2.6 (0.7–4.7)	
Rectum (*n* = 21)	49.3 (46.6–69.1)		353 (286–451)^1^		1.7 (0.7–3.8)	
TNM stage:		0.693		0.329		0.004^*^
I (*n* = 7)	88.2 (64.5–111.8)		227.4 (192.6–1203.5)		1.0 (0.1–1.3)^5,6^	
II (*n* = 15)	54.8 (47.9–85.0)		579.1 (349.6–1133.5)		1.2 (0.7–2.4)	
III (*n* = 40)	57.4 (50.1–84.1)		497.4 (280.4–939.6)		3.2 (1.0–7.1)^5^	
IV (*n* = 11)	59.2 (49.0–68.5)		359.2 (244.3–450.8)		3.7 (2.6–5.3)^6^	
T:		0.353		0.257		0.002^*^
1 + 2 (*n* = 11)	111.8 (71.8–116.9)^2,3^		1133.5 (212.6–1203.5)^4^		0.1 (0.1–0.8)^7^	
3 (*n* = 41)	54.7 (47.6–84.1)^2^		407.6 (204.3–1013.9)		1.6 (0.8–2.7)	
4 (*n* = 21)	54.6 (49.0–73.6)^3^		450.8 (349.6–687.2)^4^		2.8 (1.1–5.2)^7^	
N:		0.472		0.258		0.005^*^
No (*n* = 46)	64.1 (48.4–85.0)		569.9 (310.4–1133.5)		1.3 (0.2–2.7)	
Yes (*n* = 27)	54.7 (49.0–82.6)		407.6 (260.8–671.3)		3.7 (0.9–6.8)	
M:		0.513		0.102		0.032^*^
No (*n* = 62)	60.1 (48.4–85.0)		530.6 (300.0–1128.7)		1.4 (0.6–3.5)	
Yes (*n* = 11)	59.2 (49.0–68.5)		359.2 (244.3–450.8)		3.7 (2.6–5.3)	

T: primary tumor progression; N: lymph node metastasis; M: distant metastasis; ^*^statistically significant; ^1^ *P* = 0.023 for right colon versus rectum; ^2^ *P* = 0.044 for T1+2 versus T3; ^3^ *P* = 0.021 for T1+2 versus T4; ^4^ *P* < 0.001 for T1+2 versus T4; ^5^ *P* = 0.004 for I versus III; ^6^ *P* = 0.003 for I versus IV; ^7^ *P* = 0.001 for T1+2 versus T4.

## Abbreviations

CRC: Colorectal cancer
BMI: Body mass index
CRP: C-reactive protein
CEA: Carcinoembryonic antigen
oxLDL: Oxidized low-density lipoprotein
o-LAB: Antibodies against oxLDLs
TC: Total cholesterol
HDL-C: High density lipoprotein cholesterol
LDL-C: Low-density lipoprotein cholesterol
TG: Triglyceride
Glu: Glucose.
